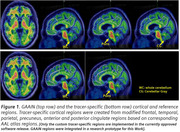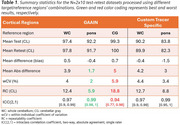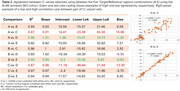# Robustness of the Centiloid scale to target and reference regions calculated with a commercial software using [^18^F]flutemetamol PET images

**DOI:** 10.1002/alz70856_107393

**Published:** 2026-01-09

**Authors:** Rachid Fahmi, Ariane Bollack, Mark R Battle, Gill Farrar, Bruce Spottiswoode

**Affiliations:** ^1^ Siemens Medical Solutions USA, Inc., Molecular Imaging, Knoxville, TN, USA; ^2^ UCL Centre for Medical Image Computing, London, United Kingdom; ^3^ University College London, London, United Kingdom; ^4^ GE HealthCare, Chalfont St Giles, Buckinghamshire, United Kingdom; ^5^ Siemens Medical Solutions USA, Inc., Knoxville, TN, USA

## Abstract

**Background:**

Quantification of amyloid‐PET using the Centiloid (CL) scale has been increasingly used in clinical trials of AD modifying therapies and is set to play a role in clinical decision‐making. We assessed the effect of target/reference regions’ combinations on repeatability, reproducibility, and reliability of the CL metric using a commercial software applied to ^18^F‐flutemetamol scans.

**Methods:**

A regulatory‐approved software was used for CL calculation using five different target/reference region (TR/RR) combinations: two custom‐made tracer‐specific TRs combined with either whole‐cerebellum (WC) or pons; and three combinations of GAAIN cortex with either GAAIN‐WC, GAAIN‐pons or GAAIN‐Cerebellar Gray (CG) (Figure 1). An AD test‐retest cohort (*N* = 10x2) was used to assess within‐subject repeatability, and an amnestic MCI cohort (*N* = 80) was used to assess reproducibility in terms of absolute agreement in continuous CL values, and reliability for dichotomizing scans into amyloid positive or negative using these thresholds: 11, 25 and 37 CL.

**Results:**

Regardless of TR, the pons resulted in CL underestimation (∼23% on average) compared to other RRs. The test‐retest analysis (Table 1) yielded absolute biases < 5CL for all region combinations, except when using CG as RR. Within‐subject coefficients of variation ranged between [2, 5.9]% and 95% repeatability coefficients from [5.9, 18.8]CL, with the pons and the CG providing the best and worst results, respectively. Moderate‐to‐strong correlations between pairs of CL values was observed, with R^2^ in [0.87, 0.99] range (Table 2), and with 95% limits of agreement between 10 and 46 CL. Worse agreements were seen for ‘pons vs. CG’ as RR, regardless of the cortical mask (GAAIN or custom). Agreement in +/‐ status between CL sets ranged from 89‐99% (kappa: 0.63‐1.0) with lowest agreement at the 11CL cutoff occurring between CG and pons results.

**Conclusion:**

Best and worst within‐subject repeatability results correspond to the pons and CG combined with GAAIN cortical mask, respectively. Overall, quantified CLs were highly reproducible across pairs of pipelines, when same RR (pons or WC) was used with either GAAIN or Custom TRs. Worst reproducibility results correspond to pons vs. CG. Reliable dichotomization agreements were obtained at 25 and 37 CL thresholds, but less so at 11CL.